# P20 An evaluation of high oral bioavailability antibiotic prescriptions in Forth Valley Royal Hospital

**DOI:** 10.1093/jacamr/dlae136.024

**Published:** 2024-08-23

**Authors:** Eilidh Urquhart, Margaret-Mary McGlone, Hannah Soulsby, Euan Proud

**Affiliations:** Forth Valley Royal Hospital, Larbert, UK; Forth Valley Royal Hospital, Larbert, UK; Forth Valley Royal Hospital, Larbert, UK; Forth Valley Royal Hospital, Larbert, UK

## Abstract

**Background:**

Metronidazole, clindamycin, levofloxacin and ciprofloxacin have high oral bioavailability. In many cases, antibiotics are administered IV when the oral route would be appropriate. There are risks of IV administration including complications of IV access and prolonged hospital admission.

**Objectives:**

To audit current prescribing practices of four antibiotics with high oral bioavailability across medical and surgical wards in Forth Valley Royal Hospital.

**Methods:**

Patients receiving selected antibiotics were identified using the electronic prescribing system. Patients in intensive care, day units and maternity wards were excluded. An audit tool was used to collect information on antibiotic indication, the ward and specialty, and whether there were any contraindications to oral administration. Contraindications included being nil by mouth, clinically unwell or sepsis, and poor gastrointestinal absorption. Data were collected over a 5 month period.

**Results:**

In total, 98 IV prescriptions were reviewed. These included metronidazole (87), clindamycin (6) and levofloxacin (5). There were no IV prescriptions of ciprofloxacin. Fifty-three (54%) IV prescriptions were for patients with no contraindication to an oral route. Of these, 37 (70%) were under the care of a surgical specialty and 15 (28%) were under the care of a medical specialty. The most common indication for metronidazole was intra-abdominal infection. A total of 45 of the 87 (52%) patients prescribed IV metronidazole had no contraindication to the oral route and 34 (76%) of these patients were under the care of general surgery.

**Conclusions:**

High oral bioavailability antibiotics were frequently prescribed IV when the oral route may have been appropriate. Both medical and surgical departments could improve the use of oral antibiotics in appropriate patients. Work is needed to promote awareness among prescribers of these high oral bioavailability antibiotics, particularly the use of oral metronidazole in appropriate surgical patients.

